# How to Improve the Physical Health in Patients with Severe Mental Disorders

**DOI:** 10.1192/j.eurpsy.2022.67

**Published:** 2022-09-01

**Authors:** P. Falkai

**Affiliations:** Ludwig-Maximilians-Univeritiät Munich, Department Of Psychiatry And Psychotherapy, Munich, Germany

**Keywords:** Mental Disorders, Treatment, exercise, physical health

## Abstract

Schizophrenia is a severe brain disorder characterised by positive, negative, affective and cognitive symptoms and can be regarded as a disorder of impaired neural plasticity. This lecture focusses on the beneficial role of exercise in schizophrenia and its underlying mechanisms.Apart from the established pharmacological treatments in schizophrenia, aerobic exercise has a profound impact on the plasticity of the brain of both rodents and humans such as inducing the proliferation and differentiation of neural progenitor cells of the hippocampus in mice and rats. Aerobic exercise enhances LTP and leads to a better performance in hippocampus related memory tasks, eventually by increasing metabolic and synaptic plasticity related proteins in the hippocampus. In healthy humans, regular aerobic exercise increases hippocampal volume and seems to diminish processes of ageing like brain atrophy and cognitive decline.Several meta-analyses demonstrate the beneficial effect of exercise on function, positive as well as negative symptoms and brain structure in multi-episode schizophrenia.

**Disclosure:**

No significant relationships.

